# The supporting role of dogs in the inpatient setting: a systematic review of the therapeutic effects of animal-assisted therapy with dogs for children and adolescents in an inpatient setting

**DOI:** 10.1007/s00787-023-02326-1

**Published:** 2023-12-26

**Authors:** Dustin Fornefeld, Undine Zellin, Peter Schmidt, Oliver Fricke

**Affiliations:** 1 Department of Special Care Dentistry, Witten/Herdecke University, Alfred-Herrhausen-Str 50, 58448 Witten, Germany; 2 Department of Child and Adolescent Psychotherapy and Psychiatry, Gemeinschaftskrankenhaus Herdecke, Herdecke, Germany; 3 Department of Artistic Therapies and Therapy Sciences, Institute for Art Therapy, Alanus University of Arts and Social Sciences, Alfter, Germany; 4 Faculty of Health, Department of Human Medicine, Witten/Herdecke University, Witten, Germany; 5 Department for Child and Adolescent Psychiatry and Psychotherapy, Klinikum Stuttgart, Stuttgart, Germany

**Keywords:** Psychiatric disorders in children and adolescents, Child and adolescent inpatient psychiatry, Canine-assisted therapy, Animal-assisted therapy, Hospital care settings, Pet therapy

## Abstract

Animal-assisted therapy (AAT) is becoming increasingly popular. The possibilities and guidelines for interventions and methods are very diverse. Currently, published studies mainly concentrate on effects in paediatrics, outpatient therapy and schools. Specific recommendations for AAT in the context of inpatient child and adolescent psychiatry do not exist. This systematic review will attempt to evaluate the existing studies in terms of their methodological quality and specify positive and negative effects, aiming to provide a decision-making aid for everyday clinical practice. A systematic literature search (PubMed/MEDLINE, APA PsycINFO, PubPsych, ProQuest, Google Scholar, and Cochrane Library) according to the PRISMA criteria resulted in 1,908 identified hits, of which 49 articles were reviewed in full text. Three raters contributed to the review of the articles using a criteria-guided codebook. This systematic review is listed in the PROSPERO database (CRD42022358909). Quality analysis was conducted using Effective Public Health Practice Project (EPHPP). Five studies were identified. The majority of these showed deficits in quality. Therapeutic effects and positive influences on the psychopathological status, interpersonal relationships and subjective well-being or attitudes towards canine-assisted therapy (CAT) could be identified. Current studies indicate positive therapeutic effects of CAT in the inpatient treatment of children and adolescents. A cautiously positive perspective is warranted, but a general recommendation for CAT cannot be given. CAT should be carefully considered, planned, and implemented by professionals. For the future, further randomised controlled studies including follow-up studies, larger subject groups and clinically evaluated interventions are necessary to validate the current results.

## Introduction

The integration of animal-assisted services into psychotherapy, coaching, counselling, and guidance is experiencing rapid growth. Since Levinson's pioneering efforts in 1969 [1] to incorporate animal-assisted interventions into psychotherapy for children and adolescents, there have been notable advancements, changes, and developmental processes. In 2011—42 years after Levinson’s initiative—animal-assisted therapy is still regarded as an "innovative method" [2] in the treating psychiatric or somatic disorders, even though numerous studies demonstrating a positive effect can be identified [2]. During this period, systematic reviews, beyond the initial favourable conclusions, have consistently critiqued the fragmented evidence concerning the intervention, alongside deliberations on how to address these concerns and what recommendations could be derived from them [3, 4].

Now, two decades later, a valid question emerges: whether animal-assisted therapy (abbreviated as AAT) or canine-assisted therapy (abbreviated as CAT) still retains its innovative status or if it has now undergone widespread scientific evaluation, becoming an integral aspect of the classical psychotherapeutic and psychiatric framework [5]. Its application span outpatient treatment [6–8], inpatient care [9–11], palliative care [12], dental visits [13] and notably, during examinations [14]. It is hard nowadays to imagine therapeutic-pedagogical work without animal-assisted work.

The incorporation of AAT and CAT extends beyond the realm of inpatient child and adolescent psychiatry; in some cases, AAT and particularly CAT form an integral component of multimodal therapy programmes. Presently, the imperative of offering the best possible treatment to children and adolescents is undeniable. How, despite its widespread practical utilisation, the question lingers regarding how the integration AAT within inpatient child and adolescent psychiatric facilities should be evaluated. This context also raises the issue of establishing a robust evidential foundation and conducting a factual risk–benefit assessment. Against this backdrop, the current systematic review aims to consolidate findings to ultimately address whether CAT exerts a therapeutic effect on inpatient child and adolescent psychiatric treatment. The results are intended to provide support to clinical practitioners in determining whether to employ or recommend CAT.

### Definition

Since 2000s, concerted efforts have been undertaken to establish a robust scientific framework underpinning the increasingly diverse landscape of AAT [15]. The biggest problem in this context is the lack of differentiation from related terms and the synonymous use of different terms. As early as 2003, there were 20 different definitions and twelve different terms in the English-speaking world, all converging around the theme of human-animal cooperation [16]. It is often a matter of small nuances in the work, for example whether work is done together with the animal (supportive) or only in the presence of the animal (accompanying) [17] Today, the terms “canine-assisted therapy” (CAT) or "Animal-Assisted Therapy" (AAT) are often used in the English-speaking world. The former (AAT) has been established by the European Society for Animal-Assisted Therapy (ESAAT) as a generally valid technical term [18]. In this context, therapy is understood as any professional helping relationship with an influence on humans and also includes preventive and supportive measures [19].

AAT invariably involves the triangular interplay amongst the client, the animal, and the therapist. It involves consciously planned psychological interventions with animals for children, adolescents, adults, and older people with cognitive, social-emotional and motor impairments, behavioural disorders, and special needs [19]. These interventions are meticulously planned and not happenstance occurrences in the presence of a Therapy Assistance Dog (TAD); instead, they necessitate precise implementation drive by pre-established indications and corresponding interventions [20]. Modalities encompass strategies that facilitate patient-animal interaction, communication aided by the animal or tasks directed towards the animal. The actual implementation of the planned intervention is always based on a clear orientation on the basis of the respective needs and resources, but also based on the patient's disorder [19]. The objectives of AAT exhibit variability contingent upon the application domain. Specific goals are set according to the basic profession, individual resources, needs and indication. However, in summary, four global goals can be identified that can be realised across disciplines: (1) restore, maintain, or build physical, cognitive, or emotional functions, (2) promote abilities and skills to perform activities and actions, (3) promote inclusion in the respective life situation, (4) improve subjective well-being [19].

### Basic findings on the effects of animal-assisted therapy

AATs have been extensively applied across a diverse spectrum of settings, including educational institutions, counselling, coaching and therapy. These interventions have undergone rigorous evaluation through empirical studies. In the following, results from clinical practice will be highlighted, as they seem to be particularly relevant for this context. Notably, systematic reviews and meta-analyses examining both CAT [21, 22] and AAT in a broader sense, which encompasses CAT [23, 24], originating from medical–therapeutic contexts outside this domain, such as general somatic care or outpatient nursing for adolescents and adults, consistently establish that AAT engenders salutary and constructive effects on stress, pain, and psychiatric ailments. One systematic review [22] will be highlighted, as the effects of CAT in outpatient adolescent psychotherapeutic treatment of adolescents (age: 10–19) have been investigated. The authors conclude that there is a therapeutic effect on symptomatology and the severity of the main disorder. However, the review underscores the imperative for further investigations to substantiate these findings. It should be noted that the above-mentioned analyses also identified studies that showed no measurable positive effects. Furthermore, certain studies exhibit methodological deficiencies of such magnitude that potential positive effects could only be assessed within constrained limits, particularly when interventions of varied forms were concurrently employed, thus obfuscating the attribution of effects to a specific intervention. The presence or absence of a measurable effect appears independent of the diagnosis [25], even encompassing factors such as whether the patient is a dog owner himself [26].

In the inpatient psychiatric treatment of adults, there are already several studies that highlight the positive effects, for example in the treatment of depression [27]. The presence of a TAD generally led to a significantly friendlier and less tense feeling on the ward [9]. When a TAD is present, patients tend to spend more time in the company of fellow patients. Moreover, this experience of joy and acceptance extends its influence on therapies wherein the TAD is not present [9]. The number of occupancy days in inpatient psychiatric facilities for adults could be significantly reduced using CAT [9, 25]. Adults benefiting from CAT report heightened social interaction skills, leading to improved social conduct and interpersonal communication [6]. Stress-related physiological parameters, including hearth rate, blood pressure and cortisol secretion, register significant reductions, especially during the interactions with TADs [12, 28, 29], subsequently fostering cardiovascular health improvements [21]. Owing to this anxiolytic effect, TADs are employed in various contexts, such as examination settings or dental treatments for individuals with anxiety disorders [13, 14]. In self-evaluation, patients report that the use of a TAD was one of the most effective factors in inpatient therapy, contributing to recovery in all areas of life, even when there was only a significant reduction in symptoms in the area of anxiety [26], which also reflects the high subjective degree of therapeutic achievements. Furthermore, notable enhancements in global psychosocial functioning and reductions in internalised problems are documented [30].

In the field of child and adolescent psychiatry, CAT has been particularly well evaluated in the outpatient treatment of children and adolescents with autism spectrum disorder [31–33]. Robust data stem from clinical trials [4, 34, 35], patient and parental surveys [36] and multiple systematic reviews [3, 4]. In both systematic reviews [3, 4] the positive impression and positive tendencies became clear, especially in the areas of social behaviour and psychosocial interaction, greater use of language and stress reduction. The TADs often assume roles as catalysts for speech or as facilitators of social initiation. However, caveats regarding methodological rigour are also acknowledged, primarily revolving around sample size constraints, lack of standardisation and protocol adherence and heterogeneity in terminologies and interventions employed.

### The uniqueness of working with therapy-assisting dogs

This work deals exclusively with the therapeutic effects of TADs. This focus is deliberate, given that TADs possess distinct skills and qualities rendering them exceptionally suitable for therapeutic purposes. Some of these distinctive capacities are linked to their potential for Theory of Mind (abbreviated as ToM). Extensive evidence supports the notion that dogs, to a considerable extent, exhibit metacognition, perspective-taking, and intention attribution—hallmarks of ToM. Dogs adeptly discern varied social cues and adeptly leverage them for their advantage [37, 38]. Perspective-taking also extends to interpreting attention states and they adeptly adjust their behaviour in response to a person’s state of attention, thereby evincing their capacity for recognition [37, 38]. From this, assumptions can be made about further social-cognitive competencies [39–43]. Funda and Dustmann [44, 45] have demonstrated that there is no discernible distinction in ToM ability between trained and untrained TADs. All dogs inherently possess this capability. Nevertheless, the special importance of criteria-based training and further education should be pointed out. Certainly, training in the field leads to acquiring skills, knowledge of methods, legal frameworks, hygiene guidelines and a criteria-based analysis of the situation and planning of further interventions.

Dogs are inherently well-suited for AAT owing their aforementioned attributes. Beyond there traits, they also enjoy a favourable reputation in Western culture. Moreover, their versatility is noteworthy, as they can be effectively utilised across diverse settings, including indoor spaces devoid of designated outdoor areas such as fields or equine stables. The distinctive nature of CAT lies in their well-defined social competencies, adaptable application modalities and the uniformly positive response evoked by the presence of the dogs.

### Aim of this systematic review

This systematic review aims to answer the following research question: Is there a significant improvement in general mental health status or measurable effects in the recovery of specific mental illnesses through the use of a therapy assistance dog in inpatient child and adolescent psychiatry compared to standard therapy?

## Methods

In PROSPERO, a protocol was published on 20th September 2022; ID: CRD42022358909. The method presented below follows the PRISMA guideline for systematic literature reviews [46].

### Eligibility criteria

In order to be included in the present systematic review, the studies investigated were required to satisfy specific criteria concerning participants, interventions, comparison groups and outcomes (PICO) [47]. Studies involving children and adolescents up to the age of 18, without known intellectual disabilities and currently undergoing inpatient treatment within child and adolescent psychiatry for mental illnesses were considered. Studies exploring CAT with children and adolescents in forensics or paediatrics were excluded. The studies had to exhibit a distinct emphasis on CAT within the overarching psychiatric-psychotherapeutic framework. This systematic review is limited to CAT because it is important to be as setting- and animal-specific as possible in the analysis and comparison of data. This approach is aimed at circumventing the confounding effects that arise from the amalgamation of various methods and animals across disparate settings, which compromises the quality and significance of studies and systematic reviews in the broader realm of AAT. Although acknowledging the limited data available, we consciously adopted a setting-, age-, and animal-specific approach to enhance precision. The measure should have been examined with recognised questionnaires or concepts. In addition, only studies with at least two measurement points and a total of more than ten subjects were included. Only journals published in English or German from the last ten years (01.01.2012–31.07.2022) were eligible. This time period seemed necessary as the scientific discourse and professionalism has changed significantly in the last ten years. Further studies were integrated by cross-referencing or citations from other studies and bibliographies.

### Search strategy

Before conducting the systematic literature review, a search strategy aligned with the PICO criteria [47] was developed. The population of children and adolescents currently undergoing inpatient psychiatric treatment was described using terms such as "child", "youth", "adoles*" or "mental disorder". Additionally, prominent mental disorders prevalent in childhood and adolescence, such as depression [48], were encompassed to augment the scope. Pertaining to the intervention, terms were selected that yielded AAT or CAT within the context of psychiatric and psychotherapeutic treatment. Terms like "animal assisted*", "canine therapy" or "therapy dog" were used. As for outcomes, the focus lay on the general mental state or symptom-related changes assessed through clinically validated questionnaires. The comparative intervention and outcome were not included in the search strategy, as it would limit the search results too much and therefore possibly relevant studies would not be included. Refer to Table 1 for comprehensive details.

**Table 1:** Application of the PICO schema to the search query at hand

Term	Short explanation	Application to the search query	Terms English	Terms German
P – patient / population	The most important characteristic of the patient in terms of age, clinical picture, condition or gender	Children or adolescents with psychiatric illnesses who are currently undergoing full inpatient psychiatric treatment	psychiatry, obsessive compulsive disorder, depression, suicide*, anxiety, impulsivity, child, youth, adolescent, adolescence, inpatient, mental disorder, acute mental disorder	Psychiatrie, Zwangsstörung, Depression, Suizid*, Angststörung, Impulsivität, Kind, Jugendlich, Pubertät, stationäre Therapie, psychische Erkrankung
I – intervention / therapie	The most important intervention in terms of medication, test or further reviews	Animal-assisted therapy as part of psychiatric-psychotherapeutic treatment	dog, therapy dog, canine therapy, animal-assisted*	Hund, Therapiebegleithund, hundegestützte Therapie, Tiergestützte Therapie
C – comparison	The most important other possible form of treatment, e.g. placebo, standard therapy	If possible, compared to standard therapy, or treatment without animal-assisted therapy	Will not be considered further, as it would limit the research results too far	Will not be considered further, as it would limit the research results too far
O – outcome	Description of what is to be achieved or measured	Mental general condition, mapped via any clinically evaluated questionnaires	Will not be considered further, as it would limit the research results too far	Will not be considered further, as it would limit the research results too far

### Data sources

For this systematic review, a wide range of national and international journals and scientific databases were searched. The international specialised databases encompassed PubMed, APA PsycInfo, Cochrane Library and ProQuest. This was supplemented by the (German) national specialist database PubPsych. Furthermore, to comprehensively compile relevant articles, Google Scholar was also employed. Due to the distinctive functioning of Google Scholar, slight adjustments to the search terms have to be made.

### Study selection

**Fig. 1:**
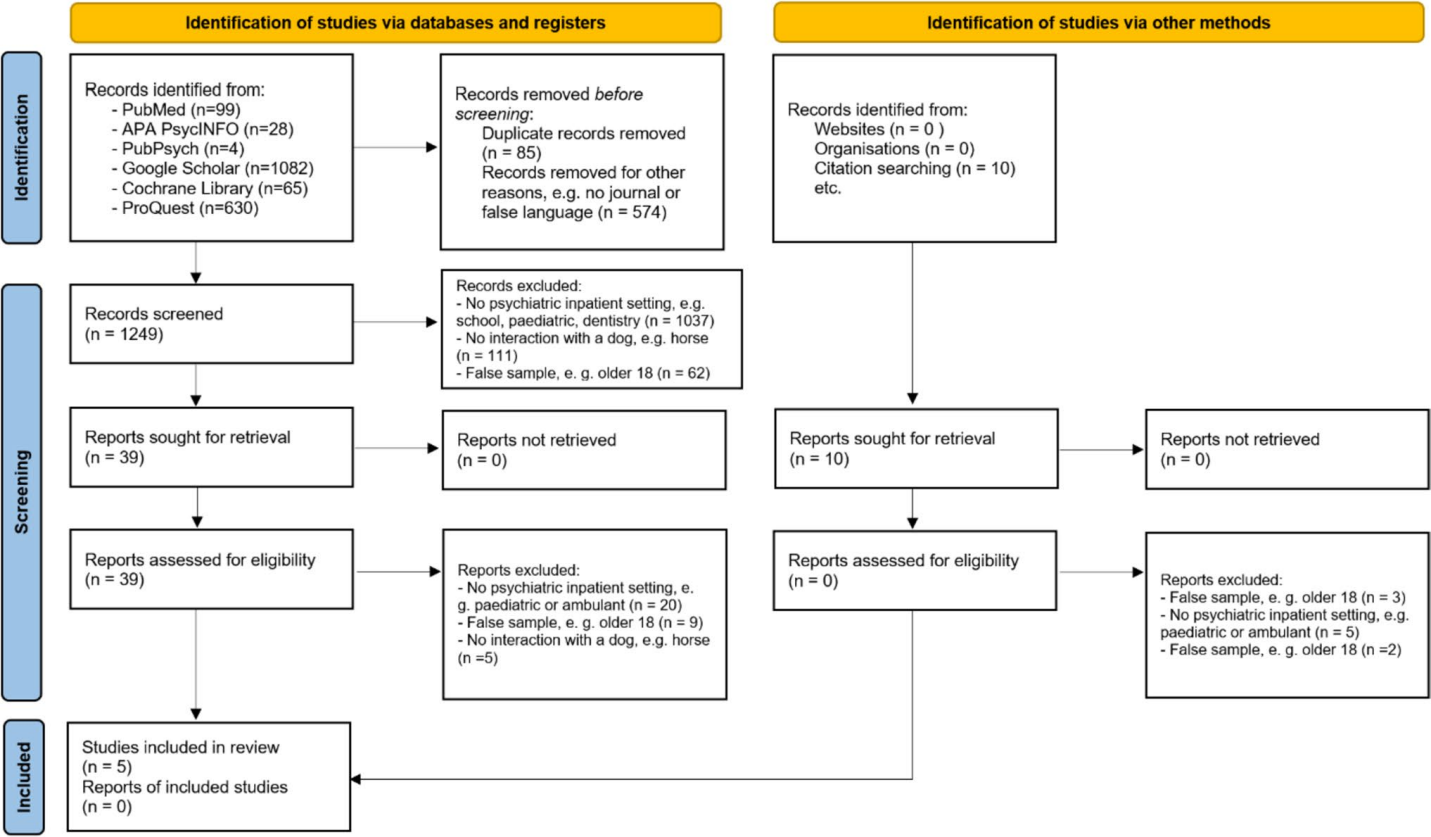
PRISMA flow diagram for the systematic review of therapeutic effects of canine-assisted therapy for children and adolescents in an inpatient setting

The results of the database searches were processed and extracted in the first step. First, the duplicates were removed, and the still unscreened articles were listed in Zotero (V6.0.22, Roy Rosenzweig Center for History and New Media, George Mason University, Washington DC). The studies were screened and selected in three stages: In the first stage, the lead author (DF) and the second author (UZ) independently screened all titles using 15 pre-defined criteria outlined in a comprehensive codebook. In this process, mainly foreign language articles (NOT German or English) or articles that had not been published in journals were rejected. In the second stage, the lead author (DF) and the second author (UZ) independently read the abstracts and evaluated them using the criteria set out in the codebook. In the final stage, both the lead author (DF) and the second author (UZ) independently engaged with the full text of the qualifying studies to ascertain their suitability for inclusion. In cases where there was uncertainty about a study, the third author (OF) was consulted to reach consensus. A summative depiction of the total count of articles, categorised according to PRISMA criteria, is visually presented in Fig. 1.

### Data extraction

The lead author (DF) and the second author (UZ) undertook the collection of data from studies that fulfilled the predetermined inclusion criteria. The following methodological information and results were evaluated and transferred into a criterion-guided overview: Aim of the study, location of the study, study design, participant details (e. g. age, gender, diagnosis), characteristics of the intervention (e. g. duration of CAT, control intervention) and type of survey (e.g. questionnaires or observation).

## Results

The literature search yielded a total of 1908 hits, of which 1249 articles remained after removing duplicates (*n* = 85) and foreign language (non-English/German) or non-scientifically published (*n* = 574). The next step was to screen the titles and abstracts. A large proportion of the studies did not align with the criterion of full inpatient psychiatric treatment (*n* = 1037). The second prevent basis for exclusion pertained to deviations from intervention criteria (*n* = 111), followed by the mismatches with the target group (*n* = 62). After this screening process of titles and abstracts, a reduced pool of 39 articles remained. These articles underwent comprehensive evaluation via full-text analysis. Moreover, additional ten articles were integrated into the full-text assessment, sourced through reference citations and contributions from domain experts. Regrettably, none of these supplementary articles were included in the review after qualified examination in the full text. Following the comprehensive review of the 39 articles, five articles emerged as fully aligned with all the pre-established inclusion and exclusion criteria.

### Descriptive overview of the studies that meet the inclusion criteria

Five studies fulfilled the inclusion and exclusion criteria in all aspects. The characteristics of the studies are summarised in Table 2. Amongst them, two originated from Italy [25; 30], whilst three hailed from the United States of America [49–51]. In all studies, the intervention under investigation took place in a full inpatient child and adolescent psychiatric facility and focussed on the therapeutic effects on the patients. Two studies also focussed on the therapeutic effect on the caregiver system and the guardians [30, 49]. The study cohorts ranged from 34 [25] to 94 [50] participants, with subjects’ average ages spanning from 11.4 [51] to 15.91 [25] years. Most studies reported a notably higher proportion of female participants, ranging from 73.4% [50] to 84.8% [51], except for one study that maintained gender balance [25]. One study, however, did not furnish comprehensive socio-demographic information [30]. The duration of interventions varied from a singular ten-minute session [51] to a pre-structured programme involving 45-min weekly sessions over a 12-week span [25]. Notably, one study omitted specification of intervention duration [30]. All studies featured CAT sessions facilitated by adept and trained professionals in conjunction with TADs. Psychological–psychiatric care was ensured through placement in the psychiatric facility. The overall length of stay in these psychiatric facilities ranged from 9.1 days [49] to an unspecified range spanning two to twelve weeks [25, 30]. However, two studies did not explicitly indicate a generalised length of stay [50, 51]. Two studies made reference to the DSM-5 classification system [50, 51], whilst one study referenced ICD-9 [25]. Two studies refrained from specifying their diagnostic basis [30, 49].

**Table 2:** Presentation of the descriptive factors of the studies; $\wedge$ significant improvement; $\to$ no significant change; $\vee$ significant decrease

Author (year)	*N* (patient/employee)	Age (avg.)	Diagnose	Study design	Comparison	AAI terminology	Intervention	Sessions (duration)	Outcome
Brown (2019) [49]	68 (50/18)	12–17 (14.85)	Depression, bipolar, anxiety, psychoses, ADHD	Quasi-experimental: pre-/post-comparison	None	Animal-assisted activity (AAA)	Petting, talking, perform tricks, interaction with the dogs handler	As long inpatient: group weekly “afternoon”	Afraid ($\vee$), sad ($\vee$); angry ($\vee$), tired ($\vee$), tense ($\vee$), energetic ($\wedge$), happy ($\wedge$)
Fodstadt (2019) [50]	94	8–17 (13.9)	Mood-, eating-, anxiety-, adjustment-, disruptive/impulse-control-, psychotic-disorder, OCD	Quasi-experimental: pre-/post comparison	None	Animal-assisted activity (AAA)	No clear structure, interaction with the dog, handler and assistant,	One time visit with parents, at least ten minutes (avg. 11.5 min)	Distress ($\vee$)
Germone (2019) [51]	44	4–17 (11.4)	ASD	Clinical repeated measurement design: cross-over	Control group: social skills group with a toy for free interaction time	Animal-assisted activity (AAA)	Free interaction time with die dogs, talk about dogs	10 min. AAA in the small group	Social-communication overall ($\wedge$), overall communication towards dog handler ($\wedge$) and towards others (→), talking towards adults ($\wedge$) and peers ($\wedge$), positive statements ($\wedge$), negative verbiage (→), absence of vocalisation (→), gestures ($\wedge$), directed eye gaze ($\wedge$), physical contact (→), prosocial behaviour (→), emotional display ($\wedge$), smiling ($\wedge$), laughing ($\wedge$), negative affect ($\wedge$), neutral facial expression ($\wedge$), over-activity behaviour ($\wedge$), aggression ($\vee$)
Stefanini (2015) [25]	34	11–17 (15.91)	Eating-, mood-, anxiety-disorder, schizophrenia	Randomised controlled trial (RCT)	Standard therapeutic protocol	Animal-assisted therapy (AAT)	Play activities, physical contact, grooming, cleaning, basic obedience, walking, agility routes	Weekly sessions over 3 months, every session: 45 min,	Format of hospital care ($\wedge$) ordinary school attendance ($\wedge$), global functioning ($\wedge$), participation ($\wedge$), socialised behaviour with adults ($\wedge$), social withdrawal behaviour ($\vee$)
Stefanini (2016) [30]	40 (20/20)	11–17	Eating, mood-disorder, schizophrenia, anxiety disorder	Cross-sectional study and RCT	Standard therapeutic protocol	Animal-assisted therapy (AAT)	Not reported	Not reported	Format of hospital care ($\wedge$), regular school attendance ($\wedge$), global functioning ($\wedge$), internalising symptoms ($\vee$), total competence ($\wedge$)

In Stefanini et al. [25], the three main diagnosis groups were eating disorders (64.7%), affective disorders with 20.6% and schizophrenia with 8.8%; 55.9% of the subjects had other mental illnesses. Fodstadt et al. [50] reported a preponderance of affective disorders (50%), followed by eating disorders (15.9%) and anxiety disorders (9.6%). In Germone et al. [51], all the subjects were diagnosed with Autism Spectrum Disorder, additionally they were attributed to anxiety disorders with 68.1%, depressive disorders with 51.1% and neurodevelopmental disorders with 29% (multiple responses possible). Brown et al. [49] did not furnish detailed diagnostic data; the primary diagnoses therein encompassed depression, bipolar disorders, anxiety disorders, schizophrenia, and ADHD.

To quantifiably assess the psychological effects of CAT, four of the five studies opted for a purely quantitative questionnaire-based survey. One study adopted a mixed-methods approach [49]. Four evaluated measurement instruments were utilised: The Children Global Assessment Scale (C-GAS; Schaffer et al. 1983), the Youth Self Report (YSR; Achenbach et al. 1994), the Subjective Unit of Distress Scale (SUDS; Wolpe 1973) and the Visual Analogue Mood Scale (VAMS). Additionally, one study incorporated video recordings and structured observations [51]. In Germone et al. [51], video recordings were analysed using OHAIRE V3 (O’Haire et al. 2013). These assessment tools were further supplemented with diverse inquiries pertaining to satisfaction with CAT, along with questionnaires encompassing hospital care and standard school attendance formats.

### Quality assessment of included studies

The Effective Public Health Panacea Project (EPHPP) quality framework was selected as the method for evaluating the quality of the studies incorporated in this analysis. The detailed results of the EPHPP quality assessment are shown in Table 3. Amongst the five studies, one study [25] attained a “Strong” rating in the Overall Ranking, signifying the absence of weak attributes within the EPHPP quality assessment. Conversely, the remaining four studies received a “Weak” rating in the Overall Ranking, signifying the presence of two or more weak quality attributes. The categories of “Study Design” and “Confounders” garnered the highest average quality ratings. Within the “Study Design” category, three studies merited a “Strong” rating. Remarkably, two studies adhered to the gold standard employing a Randomised Controlled Trial (RCT) design [25, 30]. Another study employed a clinical trial format with cross-over measurements versus a control group [51]. Two out of the five studies secured a “Moderate” rating in this category, as they encompassed pre-post measurements without a control group [49, 50]. Instances wherein a control group was utilised often lacked detailed description, merely being defined as “therapeutic protocol”. “Selection Bias” and “Blinding” emerged as the categories most prone to reflecting weak characteristics. Given that only one study achieved a “Strong” rating, and an 80% majority received a “Weak” rating, it is prudent to exercise caution when interpreting the following findings.

**Table 3:** EPHPP quality assessment method for risk of bias

Author (year)	Selection bias	Study design	Confounders	Blinding	Data collection	Withdrawal and dropout	Overall
Brown (2019) [49]	Weak	Moderate	Weak	Weak	Weak	Weak	Weak
Foodstedt (2019) [50]	Weak	Moderate	Strong	Weak	Weak	Strong	Weak
Germone (2019) [51]	Weak	Strong	Strong	Weak	Strong	Weak	Weak
Stefanini (2015) [25]	Moderate	Strong	Strong	Moderate	Moderate	Strong	Strong
Stefanini (2016) [30]	Weak	Strong	Strong	Weak	Weak	Weak	Weak

### Studies presented in this review

Two of the included studies [50, 51] utilised the above-described abilities of the TAD as an icebreaker or as a speech elicitor. In these instances, an unstructured interaction was initiated. Patients were granted autonomy to determine when and how the TAD would engage, along with the methods of approach. This allowed patients to articulate their needs and desires. The spectrum of interactions ranged from amusing patients through performing tricks to fulfilling bonding needs, such as cuddling and petting. In two studies [25, 49], interactions between humans, dogs and patients were carefully structured and pre-planned. Objectives were tailored to individual needs and deficiencies, alongside techniques and interventions necessary to achieve these goals. This encompassed physical contact to reduce stress, shared activities like agility exercises or walks, grooming tasks, such as brushing and cooperative engagement in tasks or objectives, including practising tricks together. Following the interventions, all interactions were reviewed and discussed with the patients. Regrettably, one study did not provide a precise account of how the interaction between humans and dogs was organised or the purpose of this interaction [30].

Certainly, all of the above-mentioned studies have their raison d'être, even if some of them show clear quality deficits. Hence, the ensuing results will encompass not solely the high-quality study, but also endeavour, to outline the therapeutic and supplementary impacts of CAT in the inpatient child and adolescent psychiatric milieu. This assessment will focus three dimensions: Changes in psychopathological conditions, changes in interpersonal relationships and subjective feedback on the CAT. Given the diverse array of services and study designs, this seems to be challenging, prompting frequent cross-referencing to relevant studies and frameworks. It should be noted that no long-term data are available that could be highlighted here. Therefore, only direct follow-up results are referred to.

### Initial post-intervention effects of Canine-assisted therapy on psychopathological conditions: positive changes in affective states and behaviours

Four studies examined the short-term effects of CAT on psychopathological aspects [25, 30, 49, 50]. All of these studies reported slightly favourable effects, which were discerned through clinically assessed measurement tools (e.g. C GAS, Schaffer D et al., 1983).

#### Findings in the study with a “Strong” EPHPP quality assessment overall ranking

After a continuously ongoing CAT over a longer period of time (3 months), participants exhibited a reduction in clinical severity (*t*(32) = 2.41; *p* = 0.02) and an improvement in school ability (*t*(32) = 2.25; *p* = 0.03) compared to the control group [25]. Simultaneously, their global functional level demonstrated a significant enhancement in comparison to the control group (*t*(32) = 4.57; *p* < 0.0001) [25].

#### Findings from studies with a “Weak” EPHPP quality assessment overall ranking

Subjects showed significantly lower levels of negative affect immediately following the intervention [49]. After the intervention (pre–post-comparison), adolescent participants demonstrated reduced anxiety (*z* = − 2.27; *p* = 0.02), anger (*z* = − 2.86; *p* = 0.004), fatigue (*z* = − 3.38; *p* = 0.001), sadness (*z* = − 4.02; *p* = 0.00) and tension (*z* = − 3.28; *p* = 0.001) [49]. Especially in the area of tension, this result can be supported by another study, which could highlight a significant reduction in stress and tension after the intervention (*p* = 0.000) [50]. The most prominent clinical effect of CAT appeared to be associated with the emotion of sadness [49]. Across all negatively correlated domains, subjects experienced reduced sadness post-CAT compared to pre-intervention levels (*z* = − 2.89; *p* = 0.004), concurrently reporting an increased sense of happiness (overall positive *z* = − 3.91; *p* = 0.00; happy *z* = − 4.11; *p* = 0.00) [49]. In the Youth Self Report, a significant decrease in internalising behaviours (*p* < 0.001) and an increase in resources and competences (*p* < 0.0001) could be demonstrated in the pre–post-comparison [30]. In a sample consisting of individuals with autism spectrum disorder, subjects exhibited heightened emotional expressions (e.g. facial expressions) post-intervention, surpassing expressions observed in the control group (*p* < 0.0001). Subjects displayed increased instances of smiling (*p* < 0.0001) and laughter (*p* = 0.0003), accompanied by reduced negative affect (defined as frowning or crying; *p* = 0.0042) and neutral expressions (*p* < 0.0001) [51].

### Initial post-intervention changes in interpersonal relationships (e.g. parents, employees, peers)

Four studies examine interpersonal changes [25, 30, 50, 51]. These changes can be categorised into primary therapy effects, encompassing the social behaviour of subjects and secondary therapy effects, encompassing the social behaviour of the support system or parents.

#### Primary therapy effects in the study with a “Strong” EPHPP quality assessment overall ranking

In the pre–post-comparison within the CAT group, notable improvements were observed in various domains [25]. There was heightened participation (*p* < 0.0001), increased socialised behaviour towards adults (*p* < 0.0001) and peers (*p* < 0.0001) and a significant reduction in social withdrawal behaviour (*p* < 0.04) [25]. Patients engaged in more interactions with the TAD (*p* < 0.0001) and showed more affective behaviour towards them (*p* < 0.0001) [25].

#### Primary therapy effects in the study with a “Weak” EPHPP quality assessment overall ranking

In a sample exclusively consisting of adolescents with autism spectrum disorder, significant increases in social interaction and communication were observed in the intervention group compared to the control group (*p* = 0.0001) [51]. This change relates especially to the adult handler (*p* = 0.0001) and less so towards other co-patients (*p* = 0.298) [51]. Participants showed more verbal interaction overall in the intervention group (*p* = 0.04), especially towards adults (*p* < 0.0001) [51]. There are also more gestures (*p* = 0.032) and more frequent eye contact (*p* < 0.0001), both especially towards the adult handler (*p* < 0.0001) [51]. It is worth mentioning that unlike the aforementioned RCT study, the impact on increased socialised behaviour towards adults could not be confirmed in the autistic sample (*p* = 0.0927) [51].

#### Secondary therapy effects in the studies with a “Weak” EPHPP quality assessment overall ranking

Staff members at child and adolescent psychiatric facilities subjectively reported a significant enhancement in their general mental state [49, 50]. Nonetheless, there was no significant change in clinical parameters observed within a small sample (*n* = 9) [49]. Two-thirds of the support system reported exclusively positive effects, which they subjectively attribute to their own mental state [50]. Qualitative interviews emphasised the therapeutic influence of CAT, enhancing the therapeutic environment and leading to more successful therapeutic endeavours [50]. This therapeutic effect is further illustrated by the increased acceptance and compliance of parents towards hospital care, therapeutic methods and treatment when involved in or aware of CAT. Additionally, it positively impacts the therapeutic alliance, parent behaviour and parent engagement, all of which collectively contribute to overall treatment success [30].

### Subjective feedback on the canine-assisted therapy in the studies with a “Weak” EPHPP quality assessment overall ranking

Three studies have captured the subjective feedback from participants and other individuals involved in CAT [49–51]. Qualitative statements, as well as their own subjective experience or general feedback, have been documented. These statements serve to complement and enrich the quantitative data.

Participants’ qualitative feedback largely revolves around their personal experiences. They described themselves as happier, more relaxed and calmer during and after the intervention [50]. The CAT is frequently described as joyful, enjoyable and a welcome addition to the existing therapeutic programme [50, 51]. For example, memories of their own childhood or general positive experiences with animals are stimulated [50]. Caregivers and support personnel have noticed that patients tend to open up more about their challenges and issues in the presence of the TAD. Furthermore, agitated patents often find solace and tranquillity in the company of the TAD [50]. In the Likert scale survey, a significant 77.1% of respondents expressed that they “very much enjoyed” the CAT, with no responses indicating dissatisfaction [50]. The feedback also indicates a strong attachment and affection towards the TAD [50].

The average duration of the intervention in one study was 11.5 min (SD = 3.4), which two-thirds of the respondents felt was just right. A smaller proportion of participants expressed a desire for a longer intervention period [50]. A few participants perceived the CAT as “childish” or “idiotic” [49]. No discernible difference was found between the CAT and control groups regarding negative comments about the intervention’s nature, the desire to discontinue the intervention or the wish to leave the room (*p* = 0.573) [51].

## Discussion

The aim of this systematic review was to comprehensively examine the use of CAT in inpatient psychiatric treatments for children and adolescents based on scientifically published data. The primary objective was to determine whether the inclusion of CAT in inpatient child and adolescent psychiatry leads to a significant improvement in overall mental health or measurable effect in the treatment of specific mental illnesses compared to standard therapy. In addressing this, the review investigated the impact of CAT on psychopathological, interpersonal, and subjective changes in the context of inpatient psychiatric care.

Four studies [25, 30, 49, 50] have explored the potential short-term effects of CAT on psychopathological aspects, suggesting the possibility of positive effects based on clinically evaluated measurement instruments. These potential effects included lower levels of negative effects, reduced anxiety, fatigue, sadness, and tension [49, 50]. It was suggested that CAT might have had a more noticeable clinical impact on the experience of sadness [49]. Regarding longer-term effects, there was a suggestion that CAT could potentially be associated with a decrease in clinical severity, an increase in school performance and higher global functioning levels [25]. Additionally, there were indications that CAT might have led to a modest decrease in internalising behaviour and a potential boost in resources and skills [30]. In a study involving a sample of individuals with autism spectrum disorder, there were hints that CAT might have resulted in more frequent emotional expressions [51].

These results suggest that integrating CAT into the inpatient psychiatric setting may lead to an improvement of the psychopathological conditions, particularly in the areas of mood, affect, psychomotor function, attitude towards illness, anxiety and contact behaviour [52]. A short-term improvement across six scales in the psychopathological findings is considered positive and supports the use of CAT as a therapeutic intervention in inpatient therapy alongside existing therapeutic methods. CAT, especially for patients with selective mutism or socially phobic behaviour, may serve as a useful means to encourage contact and affect in cases where they are limited. The therapeutic effect of CAT could also be beneficial for patients who exhibit agitation or impulsivity such as those with schizophrenia or individuals in acute crisis situations. However, it’s worth noting that the majority of studies did not report effect sizes due to their study designs, making it difficult to determine the magnitude of these effects. Nevertheless, considering the methodological challenges and limitations, it can be concluded that CAT in the context of inpatient child and adolescent psychiatric treatment could potentially support the success of therapy with respect to psychopathological conditions and open doors to enhance the effectiveness of other therapeutic approaches.

Four studies [25, 30, 50, 51] have examined the interpersonal changes resulting from CAT, focussing on primary and secondary therapy effects. Primary therapy effects pertain to changes in patients' social behaviour, suggesting that CAT led to increased social interaction, communication and reduced social withdrawal and aggressive behaviour [25, 51]. These findings partially support the earlier findings related to the improvement of psychopathological status, particularly in terms of contact behaviour [52], at least in interactions with peers [25, 51]. This suggests potential benefits for the treatment of socially phobic or selective mutism patients. Given the positive effect in reducing aggressive behaviour and improving psychopathological status, CAT may be a valuable addition to crisis treatment and management. Secondary therapy effects pertain to changes in social behaviour towards the helper system (e.g. parents or staff) and the reaction of the helper system to this. The evidence indicates that there is at least a short-term subjective effect on the helper system when they are integrated into CAT. The helper system believes in the therapeutic efficacy and success of CAT, considering it a valuable addition to the existing treatment programme. This belief contributes to greater compliance within the helper system, particularly amongst parents, resulting in higher treatment satisfaction and increased adherence to therapy methods and the overall hospital stay [53]. These factors positively impact the therapeutic alliance and the overall treatment success, further supporting the integration of CAT in inpatient care.

Three studies focussed on the subjective feedback of participants, encompassing both qualitative and quantitative feedback. Participants commonly reported feeling happier, more relaxed and calmer during and after CAT sessions. They viewed the interventions as joyful, enjoyable and a valuable addition to existing therapeutic programmes. This subjective feedback aligns with the scientific evaluation of positive effects, laying the groundwork for additional therapeutic benefits. Participants indicated that they were more inclined to discuss problems or life difficulties in the presence of the TAD, highlighting the potential for integrating CAT into various aspects of inpatient care, including group therapy, education, and social training programmes. One study had a mean intervention duration of 11.5 min, which was felt to be just right by over two-thirds of the participants. Due to the short duration of the intervention, the use of CAT can be implemented quickly and easily in everyday clinical practice. Only a few participants perceived the intervention as “childish” or “idiotic”. Overall, CAT was positively received by participants and was seen as providing meaningful subjective benefits.

One study documented adverse events, including two cases of patients experiencing mental decompensation with acute danger to themselves and others following CAT sessions, as well as one case of upper respiratory tract infection [50]. Causality does not seem likely to the authors of the study; however, it must be considered. All analysed studies implemented clear exclusion criteria, such as dog phobias, aversions to dogs or dog hair allergies. Patients had to provide separate consent for the intervention and be informed about less likely risks such as scratches or dog bites. It’s important to acknowledge that this intervention isn’t devoid of risks or side effects. Consequently, it is crucial to provide comprehensive information to underage patients and their legal guardians regarding all potential risks, avoiding any downplaying of such risks to ensure informed consent.

## Strengths and limitations

A strength of these systematic reviews is the rigorous PRISMA analysis and evaluation process. The review involved the assessment of 1908 articles at title and abstract level, followed by a comprehensive review of 49 full texts by two reviewers (DF, UZ). In cases of conflicts, a third reviewer with clinical experience (OF) was consulted. This multi-reviewer approach, guided by predefined scientifically sound criteria, minimised the risk of systematic bias and enhanced the quality of the literature analysis. The inclusion of several experts (DF, UZ, OF) further bolstered the credibility of the study’s result and their interpretation, thereby reducing potential errors and systematic bias.

Certain limitations are inherent to the current state of evidence. The review outlined several qualitative deficits that need further exploration. AAT is a broad field with many methodological difficulties that need to be taken into account. One limitation already exists in the heterogeneous scientific foundation and terminology. This diversity and heterogeneity are also evident in the different studies that have been presented here. Another interesting point of discussion is whether single and multiple CAT interventions are comparable or should be assessed separately. This review did not specify a minimum time limit for an intervention as an inclusion criterion. It becomes evident that comparing a one-time, approximately 11.5-min intervention to a 12-week intervention lasting several hours has clear limitations. Due to the changes in the scientific foundation of AAT, we have limited ourselves to the period of the last ten years. Certainly, there are some studies that would in principle provide noteworthy results or further supportive effects but have now been dropped from consideration. The studies mentioned above often, but not always, use valid and reliable measurement instruments. In some cases, it was explicitly noted that these instruments were not suited for use with children and adolescent. Additionally, each study used different measurement instruments, so that there was no comparability and there is not enough data for a meta-analysis at the present time. Without robust and valid measurement instruments, the significance of the collected data seems to remain diffuse and confirms the currently poor evidence base for AAT and especially CAT in child and adolescent psychiatric institutions. Meaningful measurement instruments specifically designed for evaluating AAT are completely lacking.

Many studies lack precise descriptions of test subjects, including information about age, background, and the concrete diagnosis of mental illness. Furthermore, some studies didn’t provide effect size due to their study design, making interpretation much more difficult. The studies also exhibit limitations such as small samples and a lack of control groups in certain instances. Long-term or extended follow-up studies are completely missing, which might be attributed to the relative youth of the therapy method and the inpatient setting. It should also be noted, the review includes papers from only two countries and two of the included papers are from the same research group.

## Future research

This work has identified a substantial research gap concerning the use of CAT in child and adolescent psychiatric treatment and its therapeutic and supportive effects within the inpatient therapy context. Urgent attention is needed to address this empirical data deficit through randomized controlled trials with follow-up periods of at least six months. Given the challenges of collecting and analysing such data with inpatient settings, it might be advisable to initially gather further data in outpatient or day hospital settings to establish an evidence-based foundation for CAT in outpatient care. This could potentially guide the application of findings to the inpatient setting.

In order to enable a subsequent meta-analysis, it is important that consistent, valid, and reliable measurement tools (e.g. questionnaires) should be utilised. Additionally, it’s important to determine which mental illnesses are suitable for CAT and whether any contraindications exist. For better comparability, concrete concepts and manuals should be developed and evaluated, as the heterogeneous design of CAT can lead to difficulties in comparison. It seems necessary to collect and combine clinical experiences, best practice examples and theoretically based derivations to create an evidence-based catalogue of criteria for successful CAT. Many aspects of CAT remain unclear, such as safety precautions for humans and dogs, hygiene guidelines and the careful balancing of risk and benefit [54].

## Clinical relevance

Given the identified quality deficits, the current evidence situation can be cautiously interpreted as positive. CAT could be beneficial, especially in acute settings, in cases involving internalising disorders (e.g. depression or anxiety), with children and adolescents with selective mutism (where it might serve as an “icebreaker”) or for supporting social skills training. Moreover, here are many positive studies and case reports from various medical fields, such as paediatrics and dentistry. On the other hand, the increased use of AAT, also in child and adolescent psychiatric institutions, is becoming apparent, at least in German-speaking countries, without there currently being sufficient evidence on the therapeutic effect and possible side effects or risks of this therapeutically applied method. According to the current analysis, a cautiously positive perspective is warranted, but a general recommendation for CAT in inpatient child and adolescent psychiatric facilities cannot be given at this stage. Until further research clarifies this intervention’s effectiveness, individual interventions should be carefully considered, planned, and implemented by professionals, including psychotherapists, psychiatrists, and specialists in CAT. These interventions should consider factors, such as diagnosis, compliance, and treatment planning, avoiding broad interventions without careful consideration.
